# Reducing sitting time versus adding exercise: differential effects on biomarkers of endothelial dysfunction and metabolic risk

**DOI:** 10.1038/s41598-018-26616-w

**Published:** 2018-06-05

**Authors:** Bernard M. F. M. Duvivier, Johanne E. Bolijn, Annemarie Koster, Casper G. Schalkwijk, Hans H. C. M. Savelberg, Nicolaas C. Schaper

**Affiliations:** 1 Department Human Biology and Human Movement Sciences, NUTRIM School for Nutrition and Translational Research in Metabolism, Maastricht University Medical Centre+, Maastricht, The Netherlands; 2 Department Internal Medicine, Division Endocrinology, Maastricht University Medical Centre+, Maastricht, The Netherlands; 3 Department of Social Medicine, CAPHRI Care and Public Health Research Institute, Maastricht University Medical Centre+, Maastricht, The Netherlands; 4 Department Internal Medicine, CARIM School for Cardiovascular Diseases, Maastricht University Medical Centre+, Maastricht, The Netherlands

## Abstract

Recent studies suggest that substituting sitting with light physical activity has beneficial metabolic effects, but it is unclear if this is associated with parallel changes in endothelial function. Data from three randomized cross-over studies were analyzed, in which 61 subjects (with normal weight, overweight and type 2 diabetes) followed different activity regimens (Sit, SitLess and/or Exercise) of four days each. Subjects were instructed to sit 14 h/day (‘Sit’), to substitute 1 h/day of sitting with moderate-to-vigorous cycling (‘Exercise’) or to substitute 5–6 h/day sitting with light-intensity walking and standing (‘SitLess’). Physical activity was assessed 24 h/day by accelerometry (ActivPAL) and diet was standardized. Fasted circulating biomarkers of endothelial dysfunction, lipids and insulin sensitivity were assessed the morning after each activity regimen. The endothelial dysfunction score (ED-score) was computed by averaging the Z-scores of the circulating biomarkers of endothelial dysfunction. Compared to Sit, Exercise resulted in lower ED-score, sICAM1 and sE-selectin (p < 0.05), while no significant changes were observed after SitLess. The ED-score, sVCAM1 and sE-selectin were lower after Exercise compared to SitLess (p < 0.05). In contrast, compared to Sit, insulin sensitivity (HOMA2-IR) and plasma lipids (HDL-cholesterol, non-HDL-cholesterol, total cholesterol and Apo B) did not change significantly after Exercise but were improved after SitLess (p < 0.05). In conclusion, light physical activity and moderate-to-vigorous physical activity had a differential effect on risk markers of cardio-metabolic health and suggest the need of both performing structured exercise as well as reducing sitting time on a daily basis.

## Introduction

Cardiovascular disease is one of the leading causes of morbidity and mortality in modern societies^1^. Lack of moderate-to-vigorous physical activity (MVPA) has been identified as one of the most important factors in the development of cardiovascular disease^1^. In parallel to a reduction in MVPA, sedentary behavior is also increasing^2^ and observational studies suggest that the average adult spends more than half of the waking day sedentary^3–5^. Although this is still debated^6,7^, several studies suggest that this rise in sedentary behavior might be associated with increased risk of cardiovascular disease, independent of time spent in MVPA^3,8,9^. In line with these observational studies, we recently observed that reducing sitting time and substituting it with light physical activity (LPA) improved insulin sensitivity and plasma lipids but not after MVPA, when estimated energy expenditure was held constant^10,11^. These data suggest that the negative effects of prolonged sitting on glucose and lipid metabolism cannot be fully compensated by one daily bout of MVPA but that more attention should be given to reducing sitting time. Moreover, one could also postulate that reducing sitting time by increasing LPA, such as standing or slow pace walking, might have the same beneficial cardiovascular effects as more vigorous activities, like running or cycling.

In addition to the importance of glucose and lipid metabolism for cardiovascular health, endothelial function is important as well to maintain cardiovascular health. Performing MVPA is an effective strategy to improve endothelial function^12–14^ that could be related to changes in vasoactive and/or metabolic factors such as increased shear stress and nitric oxide (NO) or altered lipoproteins and enhanced insulin sensitivity^15^. In addition to lack of MVPA, prolonged sitting has also been associated with endothelial dysfunction^16–18^. However, it remains to be elucidated, if substituting sitting with LPA affects endothelial function as well^19^. On one hand, substituting sitting with LPA improved circulating lipids and insulin sensitivity as described above^10,11^, which could have beneficial effects on the vascular endothelium^19–21^. On the other hand, it can be questioned whether engaging in LPA, like standing and low pace walking, induces sufficient cardiovascular changes and raises shear stress to such a level that endothelial dysfunction can be improved.

In recent years we performed three separate intervention studies, which had a nearly identical protocol, in which we investigated the metabolic effects of substituting sitting with LPA and MVPA in sedentary subjects with normal weight, overweight and type 2 diabetes (T2D). In this manuscript, we determined the effects of these interventions on biomarkers of endothelial dysfunction. Moreover, combining the results of these 3 randomized clinical trials enabled us to compare the differential effects of substituting sitting with LPA on biomarkers of endothelial dysfunction on one hand and metabolic risk factors on the other hand. Finally, as two of these studies also had an intervention arm with structured MVPA, we were also able to compare the effects of MVPA versus LPA on these variables.

## Methods

### Study design

Three counterbalanced randomized cross-over studies with comparable methods were carried out in which the subjects served as their own controls. The methods have been described previously in more detail^10,11,22^. The ‘normal weight study’ included healthy subjects without obesity (BMI 18–30 kg/m^2^), the ‘overweight study’ and the ‘diabetes study’ included subjects with overweight/obesity (BMI of 25–35 kg/m^2^), respectively without T2D and with T2D treated with diet only or with oral blood glucose lowering medication. All methods were carried out in accordance with the Declaration of Helsinki and all experimental protocols were approved by the Local Ethics Committee of Maastricht University Medical Centre+. All subjects provided written informed consent. The subjects were instructed to follow two or three activity regimens for four days, each in a randomized order. Between regimens, there was a wash-out period of at least ten days. In two studies, the participants were instructed to sit 14 h/day (‘Sit’ regimen), to substitute 1 h/day of sitting with cycling (‘Exercise’ regimen) or to substitute 5–6 h/day sitting with 2–4 h/day light-intensity walking and 2–3 h/day standing (‘SitLess’ regimen). In the overweight study, the participants only followed the Sit and SitLess regimen.

The activity regimens were performed under free-living conditions. The Sit and Exercise regimens differed only 1 h/day in sitting time, but the estimated energy expenditure differed significantly. The Exercise and SitLess regimens differed markedly in sitting time, but were designed to have comparable energy expenditure. To achieve a comparable daily energy expenditure during the Exercise and SitLess regimens, the intensity/duration of cycling during the Exercise regimen and the duration of additional standing/walking during the SitLess regimens were manipulated. During the Exercise regimen, approximately 1 h/day of sitting was replaced with cycling on an ergometer (Lode Excalibur, Groningen, the Netherlands) and supervised at the research centre. During the first regimen, participants carefully recorded everything they ate and drank. Subsequently, these records were returned to the participants, who were instructed to consume the same diet at the same time points during the following two regimens.

### Subjects

Subjects were recruited via advertisements at the university and in newspapers. Exclusion criteria included more than 2.5 h/week of moderate-to-vigorous intensity exercise based on self-report, diseases that interfered with physical activity participation, alcohol abuse and in the subjects without diabetes drugs that interfered with insulin metabolism. A more detailed description of the in- and exclusion criteria has been described earlier^10,11,22^.

### Assessment of physical activity

Physical activity was measured 24 h/day using an activPAL3 monitor (PAL Technologies, Glasgow, Scotland). The monitor was waterproof wrapped and attached to the skin on the anterior aspect of the thigh using Tegaderm (3 M™); non-wear was therefore not an issue. This accelerometer accurately discriminates between time spent inactive (sitting or lying), standing and walking^23^. In addition, subjects recorded their activities in a diary. This diary information was used to formulate tailor-made instructions on how to alter daily activities to guarantee optimal compliance with each activity regimen. Daily energy expenditure was estimated based on accelerometry and the heart rate/workload (wattage) during the 1 hour cycling exercise during the ‘Exercise regimen’. A more detailed description of the energy expenditure calculations has been described previously^10,11^.

### Endothelial parameters, insulin sensitivity and plasma lipids

Fasting blood was sampled via venepuncture on the morning after each 4-day regimen, at least 16 h after the last bout of cycling during the Exercise regimen. The endothelial markers analyzed were soluble Intercellular Cell Adhesion Molecule 1 (sICAM1), soluble Vascular Adhesion Molecule 1 (sVCAM1) and soluble E-selectin (sE-selectin). The analyzed metabolic markers were glucose, insulin, triglycerides, total cholesterol, high- (HDL) and low-density-lipoprotein (LDL) cholesterol, non-HDL-cholesterol and apolipoprotein B (Apo B). LDL-cholesterol was calculated using the Friedewald formula^24^. Insulin resistance was expressed as HOMA2-IR (HOMA2 insulin resistance), calculated with the use of HOMA Calculator, version 2.2.3 (The University of Oxford 2013)^25^. sE-selectin was measured with a CD62E ELISA set (Diaclone SAS, Besancon Cedex, France), a classic sandwich assay in which the analyte is captured on the relevant electrode. These captured analytes are then in turn detected by a secondary biotinylated anti-CD62E antibody. Subsequently, a streptavidin horseradish peroxidase solution and a chromogen (tetramethylbenzidine) substrate are added resulting in the progressive development of a blue colored complex. The absorbance of the color complex is then measured to accurately determine the concentration of CD62E in any sample tested. sICAM and sVCAM were measured with a sandwich multiplex-immunoassay based on electro-chemiluminescence technology (Meso Scale Discovery, Rockville MD, USA).

### Statistical analyses

To reduce biological variability, random error of each parameter and to increase statistical efficiency, endothelial dysfunction was computed by a composite score (ED-score). The ED-score consisted of sICAM1, sVCAM1 and sE-selectin with a high ED-score reflecting endothelial dysfunction^26^. The ED-scores were computed by first calculating the Z-score for each individual ((individual value − sample mean)/sample SD), with the sample mean representing the mean of one parameter over all the activity regimens; and subsequently averaging the Z-score of all the included parameters. All statistical analyses were performed with the use of IBM SPSS Statistics, version 20 (SPSS, Inc.). Numeric values were represented as mean ± standard error. Variables with a skewed distribution (sICAM1, sVCAM1, sE-selectin and HOMA2-IR) were natural logarithmic transformed before further analyses.

A mixed model analysis was used to determine interaction between the three different studies for the activity regimens. When there was no statistically significant interaction between study and regimen (p > 0.10), the data of the three studies were pooled. Subsequently, differences between the different activity regimens for the cardio-metabolic and endothelial markers were determined with a mixed model analysis and considered significantly different with a p-value ≤ 0.05. When regimens were compared pairwise, p-values ≤ 0.017 (=0.05/3, Bonferroni correction) were considered statistically significant to account for multiple testing (three pairwise comparisons). Study, regimen order, sex and activity regimen were used as fixed factors in the mixed model analysis.

### Data availability

The datasets used and/or analyzed during the current study are available from the corresponding author on reasonable request.

## Results

### Subject characteristics

**Figure 1:**
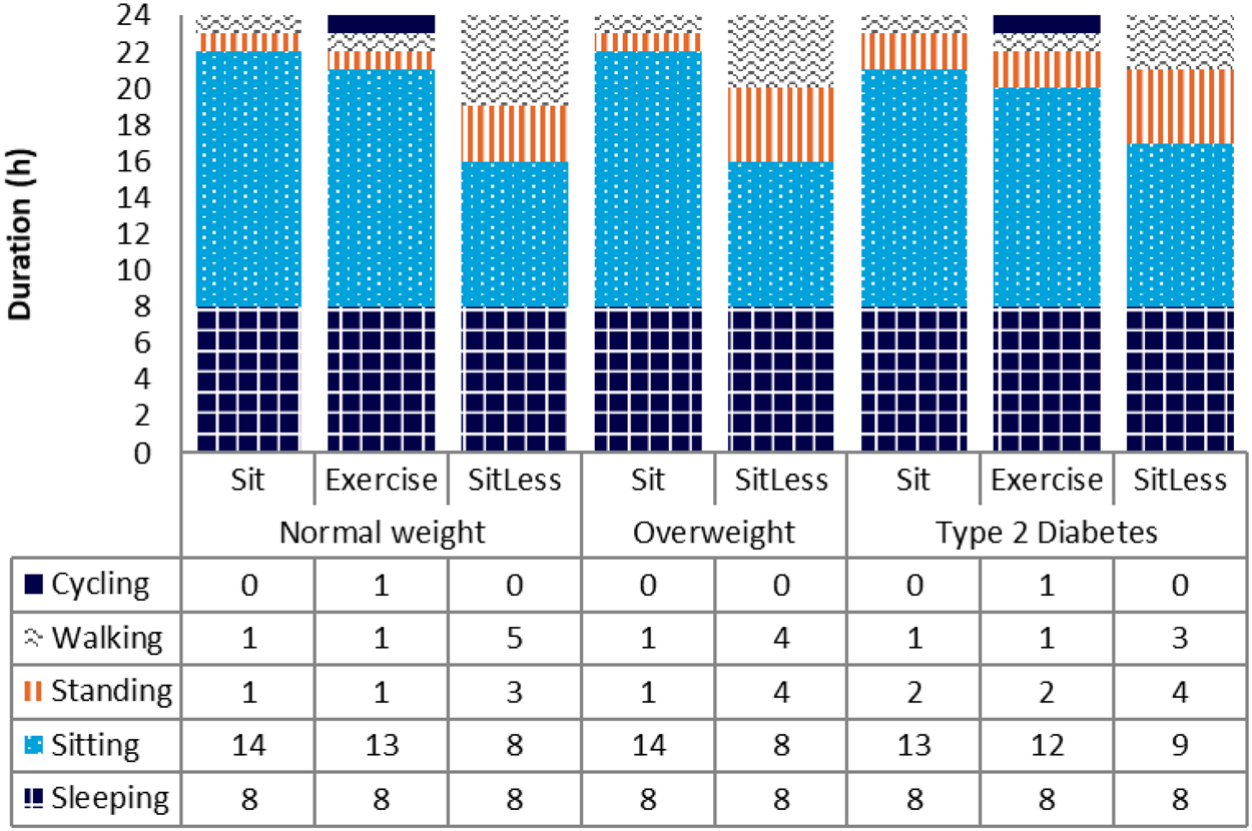
Activities performed during the activity regimens in the three different studies.

In total 65 participants were included in the three studies. Before completing the protocol, 4 participants withdrew. The subject characteristics of the remaining 61 subjects (28 male, 33 female) are described in Table 1. The participants in the normal weight study had a mean BMI of 22.6 kg/m^2^ and were on average 21 years old; none of these participants used any medication. The subjects in the overweight study had a mean BMI of 29.4 kg/m^2^ and were on average of 64 years old. The subjects in the diabetes study had a mean BMI of 30.5 kg/m^2^ and were on average 63 years old. The median (interquartile range) duration of type 2 diabetes in this study was 6 years (4–10) and diabetes was well-controlled with a mean (SD) HbA1c (Hemoglobin A1c) of 6.7% (0.8). Anti-hypertensive and lipid lowering treatment was prescribed to several subjects in the overweight as well in the diabetes study, as summarized in Table 1. The patients with diabetes were treated with oral glucose lowering medication (n = 14) or diet only (n = 5). Psychopharmaca were prescribed in 3 overweight and 1 diabetic subjects. All medication was unaltered during the interventions, except the lipid lowering medication which was discontinued 2 weeks prior to the first invention in the diabetic subjects. Physical activity (measured with the activPAL) was in accordance with the activity protocol (Fig. 1).

**Table 1:** Subject characteristics. ^A^n = 60, ^B^n = 17; ^C^n = 22; BP, blood pressure; T2 diabetes, type 2 diabetes.

Parameter	Mean ± Standard Deviation or n (%)			
	Pooled	Normal weight	Overweight	T2 diabetes
N	61	18	24	19
Sex (male/female)	28/33	2/16	13/11	13/6
Age (years)	51 ± 21	21 ± 2	64 ± 7	63 ± 9
Height (m)	1.70 ± 0.08	1.68 ± 0.07	1.72 ± 0.08	1.70 ± 0.07
Weight (kg)	80.8 ± 14.8	63.9 ± 7.8	87.1 ± 9.7	88.8 ± 12.0
BMI (kg/m^2^)	27.8 ± 4.3	22.6 ± 2.6	29.4 ± 2.3	30.5 ± 3.3
Waist circumference (cm)			104 ± 10^C^	105 ± 8
Glucose (mmol/l)	6.00 ± 1.63 ^A^	4.61 ± 0.31^B^	5.49 ± 0.61	7.88 ± 1.51
Triglycerides (mmol/l)		0.89 ± 0.25^B^		1.51 ± 0.55
Lipid lowering drugs [n (%)]	18 (30%)	0 (0%)	5 (21%)	13 (68%)
Diastolic BP (mmHg)			83 ± 9	82 ± 8
Systolic BP (mmHg)			143 ± 17	143 ± 12

### Endothelial dysfunction

**Figure 2:**
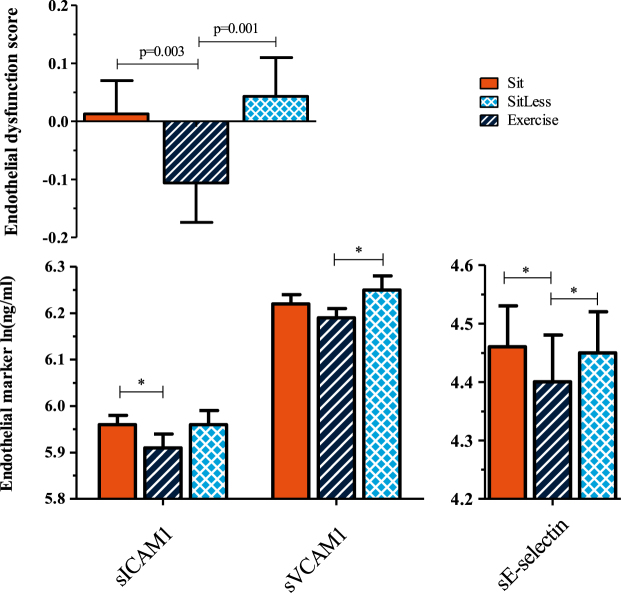
Endothelial dysfunction score and biomarkers. Data pooled for the three cross-over studies after natural log transformation, measured on the morning after each activity regimen. Means and standard error bars are presented. sE-selectin, soluble E-selectin; sICAM1, soluble Intercellular Adhesion Molecule 1; sVCAM1, soluble Vascular Cell Adhesion Molecule 1. Number of subjects: Sit = 61; SitLess = 61; Exercise = 37. *p ≤ 0.017.

In comparison to Sit, Exercise resulted in a significantly lower ED-score (p = 0.003); also the individual ED markers sICAM1 (p = 0.010) and sE-selectin (p = 0.010) were lower after Exercise compared to Sit. In contrast, when Sit and SitLess (i.e. standing and light walking) were compared, no significant differences were observed in the ED-score or individual ED markers between the two regimens. When the Exercise and SitLess regimens were compared, the ED-score was lower in the Exercise regimen (p = 0.001; Fig. 2; Additional file 1) and also sVCAM1 (p = 0.003) and sE-selectin (p = 0.011) were significantly lower after Exercise compared to SitLess (Fig. 2).

### Insulin sensitivity and plasma lipids

**Figure 3:**
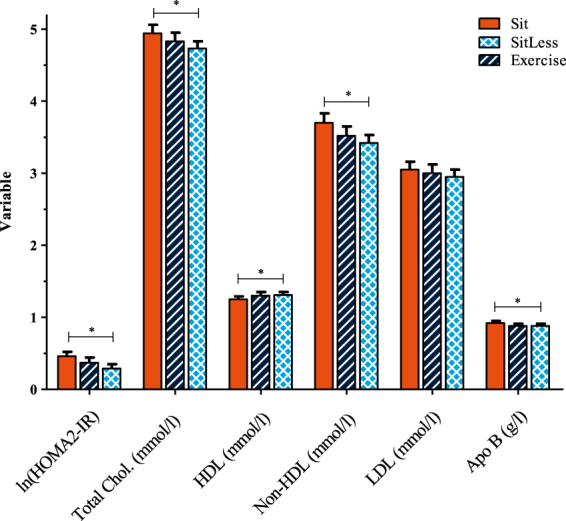
Insulin sensitivity and fasting circulating lipids. Data pooled for the three cross-over studies, measured on the morning after each activity regimen. Means and standard error bars are presented. *p ≤ 0.017; Apo B, apolipoprotein; Chol., Cholesterol; HDL, high density lipoprotein cholesterol; HOMA2-IR, HOMA2 insulin resistance; LDL, low density lipoprotein cholesterol. Number of subjects: Sit = 61; SitLess = 61; Exercise = 37.

No significant differences were observed in insulin sensitivity and plasma lipids (total cholesterol, HDL-cholesterol, non-HDL-cholesterol and Apo B) between Sit and Exercise. In contrast, all these variables were improved when SitLess was compared with Sit (p ≤ 0.017; Fig. 3; Supplemental Table 1). When the Exercise and SitLess regimens were compared, no differences were observed in all these aforementioned variables. Since response in triglycerides to the activity the regimens differed between the studies, these data could not be pooled. Triglycerides were significantly lower after SitLess in comparison to Sit in all three studies (p ≤ 0.01), and significantly lower after Exercise in comparison to Sit in the diabetes study (p ≤ 0.01) but not in the normal weight study. No differences were observed in triglyceride level between SitLess and Exercise in the latter two studies.

## Discussion

We observed that a four-day period of daily structured 60 min of MVPA improved circulating markers of endothelial dysfunction more than substituting sitting with 5–6 h/day of LPA. In contrast, substituting sitting with LPA improved circulating lipids and HOMA insulin resistance while no effect was observed of MVPA, under approximately equicaloric conditions. Our novel results suggest that daily structured MVPA can improve endothelial dysfunction despite a high sitting time (13 h/day), and vice versa that reducing sitting time by substituting it with LPA has more beneficial metabolic than endothelial effects.

A physical inactive lifestyle has been associated with several ‘classic’ cardiovascular risk factors such as increased blood pressure, central obesity, inflammation, endothelial dysfunction and insulin resistance^27^. Vascular endothelial dysfunction, which is accompanied by a reduced NO bioavailability and increased expression of vascular cell adhesion molecules like sICAM1, sVCAM1 and sE-selectin, is a key event in the subsequent development of cardiovascular risk^28^. MVPA increases NO bioavailability and alters sympathetic tone^29^. The increase in NO production is the consequence of blood flow-induced shear stress during MVPA which activates endothelial NO synthase and which subsequently attenuates the expression of adhesion molecules^30^. Our data suggest that LPA did not affect endothelial dysfunction, probably because these activities had too small effect on vascular shear stress. These results are in line with another recent study^19^ which did not observe an improvement in flow-mediated dilatation when prolonged sitting was interrupted with LPA breaks. However, the authors reported that LPA did prevent a decline in flow-mediated dilatation compared to prolonged sitting. Our results suggest that, on a short-term basis (4 days), intermittent increases in shear stress during MVPA seem to be more important for endothelial function than the improvement in insulin sensitivity as observed after LPA. However, as shear stress was not measured in our experiments, this hypothesis remains to be further explored. Our results are supported by another study in untrained women in which endothelial markers (sICAM1 and sVCAM1) were significantly lower after vigorous-intensity swimming compared to swimming at a low-intensity of longer duration^31^.

In line with earlier studies^32–36^, substituting sitting with LPA resulted in marked improvements in insulin sensitivity and lipid parameters in the pooled analysis of our experiments, as also reported for each of the individual studies previously^10,11,22^. In contrast, structured MVPA did not result in significant improvements in insulin sensitivity and most lipid variables in our analyses, while endothelial markers did improve, as discussed above. Only in subjects with T2D but not in healthy subjects, we observed an improvement in triglycerides after the exercise regimen. Previous studies have clearly indicated that MVPA can improve the aforementioned cardiovascular risk factors, but most studies did not take sitting time during the rest of the day into account. In our experiments sitting time was very high in the MVPA intervention arms (approximately 13 h/day sitting) possibly mitigating the positive effects of MVPA. Given high inter-individual differences in time spent sitting and LPA in the general population^37^, it is important to control for these variables when the effect of MVPA is investigated. For instance, when older-aged adults performed daily MVPA in line with current guidelines, total daily energy expenditure remained unaltered as the increase in energy expenditure by MVPA was compensated with longer periods of physical inactivity^38^. We measured physical activity 24 h/day and our study design is unique in that we manipulated sitting time and energy expenditure, independent of each other. During the SitLess regimen, estimated energy expenditure was comparable with the Exercise regimen, but sitting time was much lower. During the Exercise regimen, energy expenditure was high but sitting time was high as well. The Exercise regimen is reflective for the physical activity pattern of a “sedentary exerciser”, for instance an office worker who meets the physical activity guidelines by exercising 30–60 min/day but is sedentary during the rest of the day. The SitLess regimen was reflective for a “physically active non-exerciser”, for instance a salesclerk that spends many hours on his feet during working hours but does not engage in structured exercise. To our knowledge, our results are the first to show that structured exercise and reducing sitting time have a differential effect on risk markers of endothelial and metabolic health. Our findings suggest the need of both performing structured exercise as well as reducing sitting time on a daily basis in order to optimize cardiovascular health.

Strengths of our study include the strict adherence to the activity regimens in free-living conditions, which was objectively measured 24 h/day with accelerometry. Diet was standardized, and energy intake and macronutrient percentage did not differ between the activity regimens. Moreover, by combining three studies with a nearly identical study protocol, we were able to study three different populations (healthy volunteers, overweight individuals without diabetes and overweight type 2 diabetic subjects), increasing the generalizability of our results. Limitations of our study include the assessment of energy expenditure. Energy expenditure was indirectly estimated by accelerometry and heart rate/workload of cycling during the ‘Exercise’ regimen. In addition to endothelial plasma markers, future studies also need to investigate other markers of endothelial function such as flow-mediated vasodilatation of the brachial and femoral arteries. Since the volume of activities in our studies was high, the volume of LPA that is feasible in daily life also needs to be determined. An additional limitation was that the obese subjects only followed the Sit and the SitLess regimen, but not the Exercise regimen, this misbalance could have affected the power to detect differences. Finally, as our study consisted of relative short-term interventions, follow-up studies are needed to determine the long-term effects and clinical relevance of LPA on cardiovascular risk.

## Conclusion

In summary, our results suggest that LPA and MVPA have different effects on vascular and metabolic health. On one hand, reducing sitting time by increasing LPA seems important for insulin sensitivity and plasma lipids. On the other hand, MVPA had more beneficial effects than LPA on circulating endothelial parameters. To our knowledge, our results are the first to show that structured exercise and reducing sitting time have a differential effect on risk markers of cardio-metabolic health and suggest the need of both performing structured exercise as well as reducing sitting time on a daily basis.

## Electronic supplementary material

Supplemental Table 1.
