# Supplementary material for: Reducing sitting time versus adding exercise: differential effects on biomarkers of endothelial dysfunction and metabolic risk
Source: Sci Rep. 2018 Jun 5;8:8657. doi: 10.1038/s41598-018-26616-w (PMC5988819; doi:10.1038/s41598-018-26616-w)
Supplement: Supplementary file 1 — Supplemental Table 1. [file 41598_2018_26616_MOESM1_ESM.pdf]

Supplementary information

Reducing sitting time versus adding exercise: differential effects on biomarkers of endothelial dysfunction and metabolic risk

Bernard M.F.M. Duvivier[1,2*], Johanne E. Bolijn[1], Annemarie Koster[3] , Casper G. Schalkwijk[4], Hans H.C.M. Savelberg[1], Nicolaas C. Schaper[2,3,4]

1. Dept. Human Biology and Human Movement Sciences, NUTRIM School for Nutrition and Translational Research in Metabolism, Maastricht University Medical Centre +, Maastricht, The Netherlands. 2. Dept. Internal Medicine, Division Endocrinology, Maastricht University Medical Centre +, Maastricht, The Netherlands. 3. Department of Social Medicine, CAPHRI Care and Public Health Research Institute, Maastricht University Medical Centre +, Maastricht, The Netherlands. 4. Dept. Internal Medicine, CARIM School for Cardiovascular Diseases, Maastricht University Medical Centre +, Maastricht, The Netherlands. Correspondence and requests for materials should be addressed to B.M.F.M.D. (email: bernard.duvivier@maastrichtuniversity.nl).

Supplemental Table 1. Effects of the activity regimens on endothelial markers, insulin resistance and circulating lipids. [A]Analyzed after natural logarithmic transformation; [B]n=60. Column five represents the level of statistical significance (p≤0.05) between the activity regimens. Pairwise comparisons between the regimens (column six to eight) were considered significantly different after Bonferroni correction (p≤0.017). Apo B, apolipoprotein B; chol., cholesterol; ED-score, endothelial dysfunction score; Exe, Exercise; HOMA2-IR, HOMA2 insulin resistance; HDL, high density lipoprotein; LDL, low density lipoprotein; sE-selectin, soluble E-selectin; sICAM1, soluble Intercellular Adhesion Molecule 1; Sit, Sitting; SL, Sit Less; sVCAM1, soluble Vascular Cell Adhesion Molecule 1.

Parameter	Estimated Mean±SEM			P-value	98.33% Confidence Interval		
	Sit	Exe	SL		Exe-Sit	SL-Sit	Exe-SL
sICAM1[A] (ng/ml)	5.96±0.02	5.91±0.03	5.96±0.03	0.024	-0.094; -0.004	-0.044; 0.039	-0.095; 0.003
sVCAM1[A] (ng/ml)	6.22±0.02	6.19±0.02	6.25±0.03	0.008	-0.067; 0.006	-0.014; 0.082	-0.116; -0.014
sE-selectin[A] (ng/ml)	4.46±0.07	4.40±0.08	4.45±0.07	0.014	-0.130; -0.005	-0.067; 0.044	-0.108; -0.004
ED-score	0.01±0.06	-0.04±0.07	0.11±0.07	0.002	-0.211; -0.026	-0.069; 0.130	-0.257; -0.041
HOMA2-IR[A]	0.46±0.06	0.37±0.07	0.29±0.06[B]	0.012	-0.214; 0.048	-0.307; -0.022	-0.004; 0.168
Total chol. (mmol/l)	4.94±0.12	4.83±0.12	4.73±0.10	0.003	-0.329; 0.094	-0.362; -0.065	-0.079; 0.272
HDL-chol. (mmol/l)	1.25±0.04	1.30±0.05	1.31±0.04	0.025	-0.023; 0.127	0.006; 0.117	-0.084; 0.064
Non-HDL-chol.(mmol/l)	3.70±0.13	3.52±0.13	3.42±0.11	<0.001	-0.381; 0.026	-0.395; -0.156	-0.063; 0.259
LDL-chol. (mmol/l)	3.05±0.11	3.00±0.12	2.95±0.10	0.100	-0.252; 0.139	-0.235; 0.220	-0.101; 0.201
Apo B (g/l)	0.92±0.03	0.88±0.03	0.88±0.03	0.002	-0.094; 0.015	-0.072; -0.015	-0.049; 0.058
